# Moyamoya Disease in a Middle-Aged Hispanic Woman: A Case Illustration

**DOI:** 10.7759/cureus.9101

**Published:** 2020-07-09

**Authors:** Donya Bani Hani, Sami Rabah, Khaled Alabdallah, Mohammad Aldiabat, Ayah Megahed

**Affiliations:** 1 Internal Medicine, Lincoln Medical Center, New York, USA; 2 Diagnostic Radiology, Bridgeport Hospital-Yale New Haven Health Care, Bridgeport, USA

**Keywords:** moyamoya disease, moyamoya syndrome, cta, mri, stroke

## Abstract

Moyamoya disease is a rare cerebrovascular disease of unknown etiology, once known to be rare in the United States as compared to East-Asian countries, it is now an increasingly recognized cause of strokes in the United States, as the prevalence of the disease appears to be increasing. We describe a case of a 41-year-old Hispanic female patient presenting to our hospital with a stroke. She had two episodes of right arm weakness and clumsiness prior to presentation to the hospital that had resolved upon arrival. Despite a CT head negative for stroke, further imaging work-up was performed including MRI of the brain with magnetic resonance angiography (MRA) and conventional angiogram, which showed characteristic imaging findings leading to the diagnosis of Moyamoya disease. The patient subsequently underwent elective surgical intervention with Encephaloduroarteriosynangiosis (EDAS) procedure to prevent further complications.

## Introduction

Moyamoya is a rare vasculopathy that affects the internal carotid arteries of the Circle of Willis resulting in progressive stenosis and formation of collaterals known as moyamoya vessels [1]. The name comes from the Japanese word “Moyamoya” which translates to “puff of smoke” describing the collateral blood vessels characteristic of the disease [1]. Although the disease is known to have a high prevalence among people of East Asian descent (6.03 per 100,000 in Japan), the prevalence of Moyamoya disease appears to be on the rise in the United States (from 1988 to 2004, 2,247 hospital admissions for Moyamoya disease were recorded compared to 7,473 admissions from 2005 to 2008 which represents a four-fold increase in the prevalence of Moyamoya disease in the United States) [2,3]. Moyamoya disease can present with either ischemic or hemorrhagic symptoms. It appears that clinical presentation varies according to age and geographical region [4,5]. We describe a presentation of Moyamoya disease with characteristic imaging findings in a middle-aged Hispanic woman in the United States.

## Case presentation

**Figure 1:**
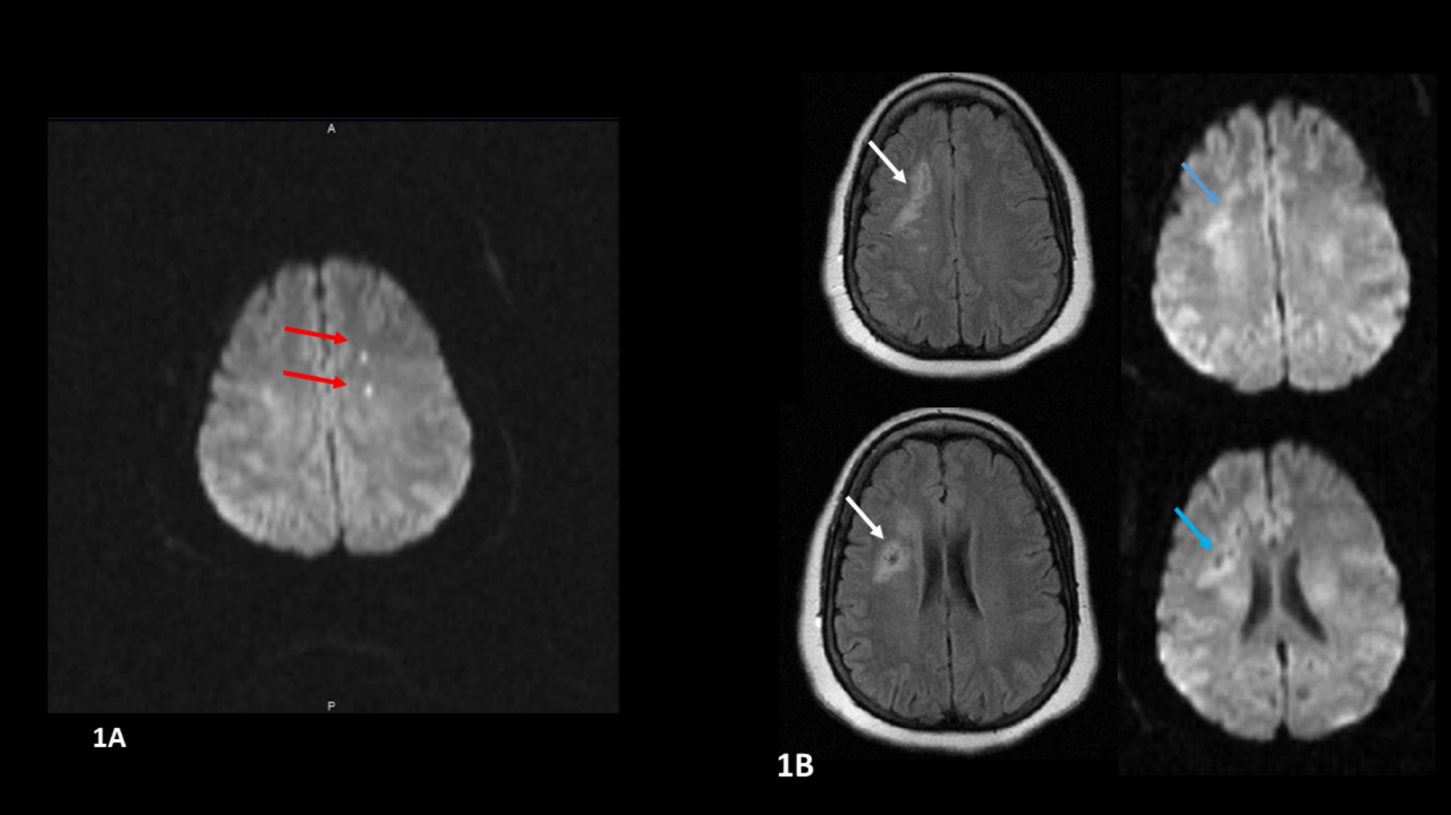
MRI of the brain MRI of the brain was done for further assessment. Figure 1A shows two foci of diffusion restriction on the diffusion-weighted sequences in the left frontal centrum semiovale consistent with acute ACA/MCA watershed infarction. Figure 1B (left: FLAIR sequences, right: diffusion-weighted sequences) shows areas of gliosis/encephalomalacia in the right frontal white matter (white arrows) with no equivalent diffusion restriction (blue arrows). Abbreviations: FLAIR, fluid attenuation inversion recovery; ACA/MCA, anterior cerebral artery/middle cerebral artery

A 41-year-old Hispanic female with a past medical history of hypertension, hyperlipidemia, and obesity presented to the hospital with complaints of two episodes of right arm weakness and clumsiness separated by a five-hour duration, each episode lasting 5 to 10 minutes. These symptoms had resolved prior to arrival at the hospital. Upon examination, she was alert and oriented, with intact motor power (5/5) in her upper and lower extremities, without any sensory loss. Her cranial nerves were grossly intact and she had a normal gait. Initial CT of the head without contrast revealed an asymmetric area of hypodensity in the right anterior frontal subcortical white matter. These findings were incongruent with the neurological symptoms the patient had described. The decision was made to pursue further imaging. Brain MRI without contrast showed two small foci of restricted diffusion within the left frontal centrum semiovale along the middle cerebral artery/anterior cerebral artery (MCA/ACA) border zone, consistent with acute watershed infarctions (Figure 1A). Areas of gliosis/encephalomalacia within the right frontal subcortical white matter were also seen, consistent with chronic infarcts involving cortical penetrating branches of the right MCA (Figure 1B).

**Figure 2:**
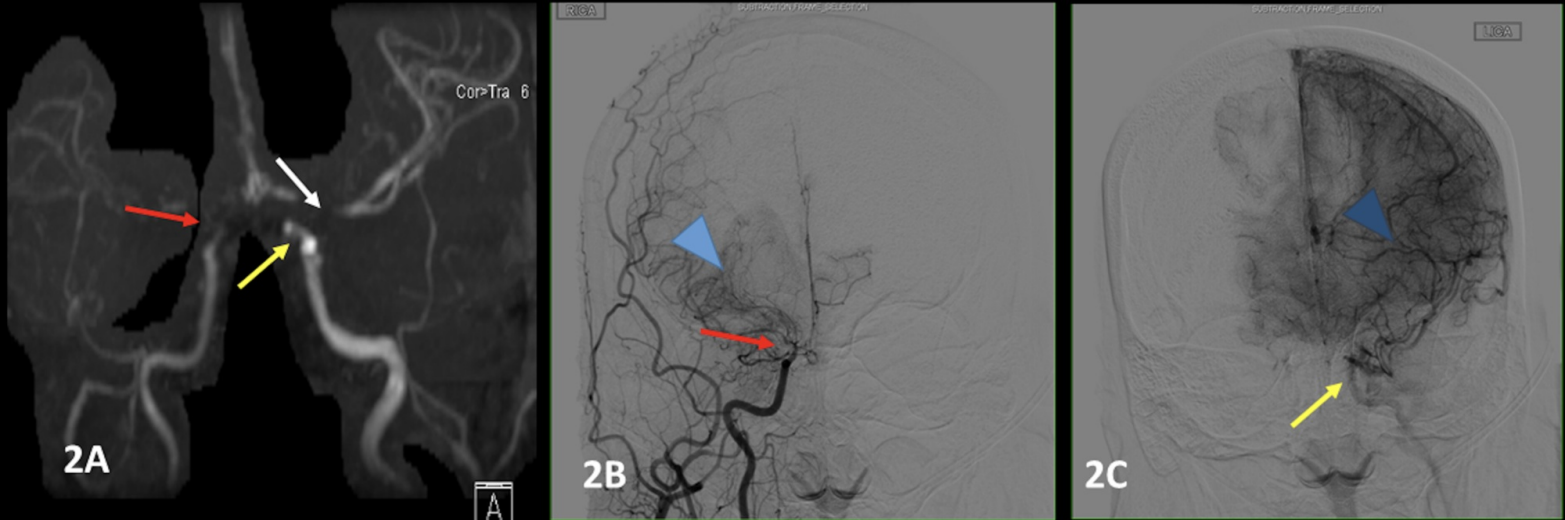
MRA and DSA Figure 2: Magnetic resonance angiography showing occlusion of the right distal ICA (red arrow), and stenosis of the distal left ICA (yellow arrow), as well as stenosis of the M1 segment of the left MCA (white arrow) (A). Digital subtraction angiography of the right ICA confirmed the occlusion of the right clinoid and supraclinoid ICA ( red arrow) and showed multiple collateral supply giving the characteristic "puff of smoke" (blue arrowhead) (B). DSA of the left ICA confirmed the occlusion of the distal left ICA ( yellow arrow) and showed a network of collateral vessels to supply the brain (dark blue arrowhead) (C) Abbreviations: DSA, digital subtraction angiography; ICA, internal carotid artery; MCA, middle cerebral artery

MRA of the head was done revealing occlusion of the distal right internal carotid artery (ICA), stenosis at the distal left ICA and occlusion of the M1 segment of the left MCA, along with Moyamoya type collaterals bilaterally, consistent with Moyamoya disease (Figure 2A). Conventional digital subtraction angiography (DSA) was done confirming the diagnosis and showing occlusion of the clinoid and supraclinoid right ICA and the characteristic “puff of smoke Moyamoya” collaterals, as well as stenosis in the distal left ICA, with multiple vascular collateral networks (Figure 2B, C). It was decided by the care team that the patient will benefit from revascularization surgery to prevent further ischemic strokes, The patient was discharged with the plan of an elective Encephaloduroarteriosynangiosis (EDAS) procedure as this was the preferred surgery at the specialty center. The surgery was done successfully three months later with no complications and no further neurological sequelae.

## Discussion

Moyamoya disease is a unique cause of cerebrovascular stroke, its clinical course differing from one geographical region to another [3]. Initially described in populations of Asian heritage with a higher prevalence in the East-Asian population as compared to the United States, it is now known to be present worldwide with an incidence of 0.086 per 100,000 in the western United States [6]. However, the prevalence of the disease appears to be increasing [2,3].

The cause of Moyamoya disease is still unknown, but genetics play an important role in its occurrence, with approximately 10% to 20% of the cases being familial [7]. Moyamoya disease differs from Moyamoya syndrome in that the latter occurs in association with other conditions such as Down syndrome, systemic lupus erythematosus (SLE), neurofibromatosis 1, and hyperthyroidism [8]. There are many different clinical presentations of the disease depending on the etiology. It can present as an ischemic stroke, transient ischemic attack (TIA), or it can present as intracranial hemorrhage as a result of rupture of the collateral vessels formed in compensation to ischemia [4]. The incidence of each presentation varies according to age and population. Multiple studies have shown that among adults of the Asian population, as compared to the US population, the predominant presentation was intracranial hemorrhage, while this appears to differ in the western population who were noted to have a higher incidence of ischemic presentation with a benign course consistent with the patient described above [3,5]. There appears to be evidence suggesting that a different phenotype of the disease exists outside of Asia, which could be a contributing factor to the difference in presentation [9].

Our patient had multiple imaging tests which met all the diagnostic criteria for Moyamoya disease. The diagnosis of Moyamoya disease is confirmed by multiple diagnostic criteria on imaging [8]. These are stenosis/occlusion of the ICA, MCA and/or ACA; abnormal networks of vasculature in the vicinity of the stenotic lesions; and the presence of these findings bilaterally. Ideally, a DSA cerebral angiogram is the gold standard for diagnosis; however, if similar findings are present on MRA, it would be sufficient for diagnosis [8].

In one study in North America, it was found that upon long-term follow-up, patients managed conservatively showed a higher rate of recurrent strokes, with an annual ischemic and hemorrhagic stroke incidence rate of 13.3% and 1.7%, respectively [10]. No definitive reversible treatment of Moyamoya disease has been found and management focuses on surgical revascularization in an attempt to reduce the risk of developing ischemic strokes or TIA in the future. Two methods of revascularization are used: direct and indirect. Our patient had an indirect EDAS procedure, but choosing between these two methods is a matter of debate. Some centers prefer indirect methods such as EDAS because of the simpler technique and lower rate of complications [11].

## Conclusions

Moyamoya disease is a rare vasculopathy that appears to be on the rise in the United States. Our case highlights the typical presentation of Moyamoya in middle-aged women in North America. More studies are needed outside of Asian countries to elucidate the regional differences in the presentation and prevalence of this rare disease.
